# Gastroesophageal junction Paneth cell carcinoma with extensive cystic and secretory features – case report and literature review

**DOI:** 10.1186/s13000-018-0775-z

**Published:** 2019-01-08

**Authors:** Wenyi Luo, Wayne L. Hofstetter, Dongfeng Tan

**Affiliations:** 1 Department of Pathology, MD Anderson Cancer Center, Houston, USA; 2 Department of Thoracic and Cardiovascular surgery, MD Anderson Cancer Center, Houston, USA

**Keywords:** Paneth cell carcinoma, Cystic, Secretion, Beta-catenin, Her-2/neu

## Abstract

**Background:**

Carcinomas composed predominantly or purely of malignant Paneth cells were rarely reported in gastrointestinal system. They have not been reported at gastroesophageal junction nor has the association with Barrett esophagus been explored. None of the previous studies has mentioned any peculiar histologic features other than typical adenocarcinoma containing neoplastic Paneth cells. The Her2/neu status and the expression of beta-catenin in Paneth cell carcinoma at gastroesophageal junction have not been studied although the activated beta-catenin pathway was recently demonstrated in neoplastic Paneth cells in colon.

**Case presentation:**

A 70-year-old Caucasian male who initially presented in the emergency room due to upper gastrointestinal bleeding was subsequently found to have a submucosal nodule at gastroesophageal junction. A diagnosis of adenocarcinoma was rendered on biopsy. Histologic examination of the subsequent endoscopic mucosal resection revealed an adenocarcinoma with various levels of differentiation which are zonally distributed. The deeper portion of the tumor showed well-differentiated bland-appearing glands with extensive cystic and secretory changes. The cytoplasm of tumor cells and secretion demonstrated marked reactivity with lysozyme antibody on immunohistochemical stain. The tumor had a peculiar Her2/neu staining pattern with cytoplasmic and nuclear stain in poorly-differentiated area and no stain in well-differentiated area. Only membranous stain was detected with beta-catenin antibody.

**Conclusion:**

We reported the first case of Paneth cell carcinoma at gastroesophageal junction. The tumor had well-differentiated area which, when sampled in small biopsies, can mimic benign lesions including those related to proton pump inhibitor therapy. Lysozyme immunohistochemical stain may be helpful when difficulty in diagnosis arises. Her-2/neu was negative but showed a distinct staining pattern. In contrast to neoplastic Paneth cells in colon, beta-catenin pathway did not seem to be activated. More studies are needed for the etiology, pathogenesis, clinical course, prognosis and treatment of Paneth cell carcinoma.

## Background

Paneth cells are unique epithelial cells located at the crypt base of small intestine and proximal colon that play a key role in defense against a broad range of intestinal microbes and in the regulation of host immunity by secreting the antimicrobial alpha defensin peptides HD5 and HD6 as well as enzymes including lysozyme and phospholipase A2 [1].

Paneth cell metaplasia is a well-known phenomenon especially in chronic inflammatory conditions of gastrointestinal tract. It is considered as a sign of chronicity and has been attributed to the effect of repair and regeneration [2]. Recent publication demonstrated more frequent *K-ras* mutation and loss of heterozygosity of microsatellite markers in colonic Paneth cell metaplasia, suggesting Paneth cell metaplasia as a pre-neoplastic lesion.

Carcinomas composed predominantly or purely of malignant Paneth cells are rarely reported in gastrointestinal system. Little is known about the relationship between Paneth cell metaplasia and Paneth cell carcinoma nor have any precursor lesions been described. The activated Apc/beta-catenin/Tcf pathway was recently demonstrated in neoplastic Paneth cells in colon, suggesting a different mechanism from Paneth cell metaplasia might exist. Here we reported a case of Paneth cell carcinoma with peculiar morphology and Her2/neu staining pattern at gastroesophageal junction. The possible pathogenesis and clinical implications are discussed.

## Case presentation

### Clinical history

**Fig. 1:**
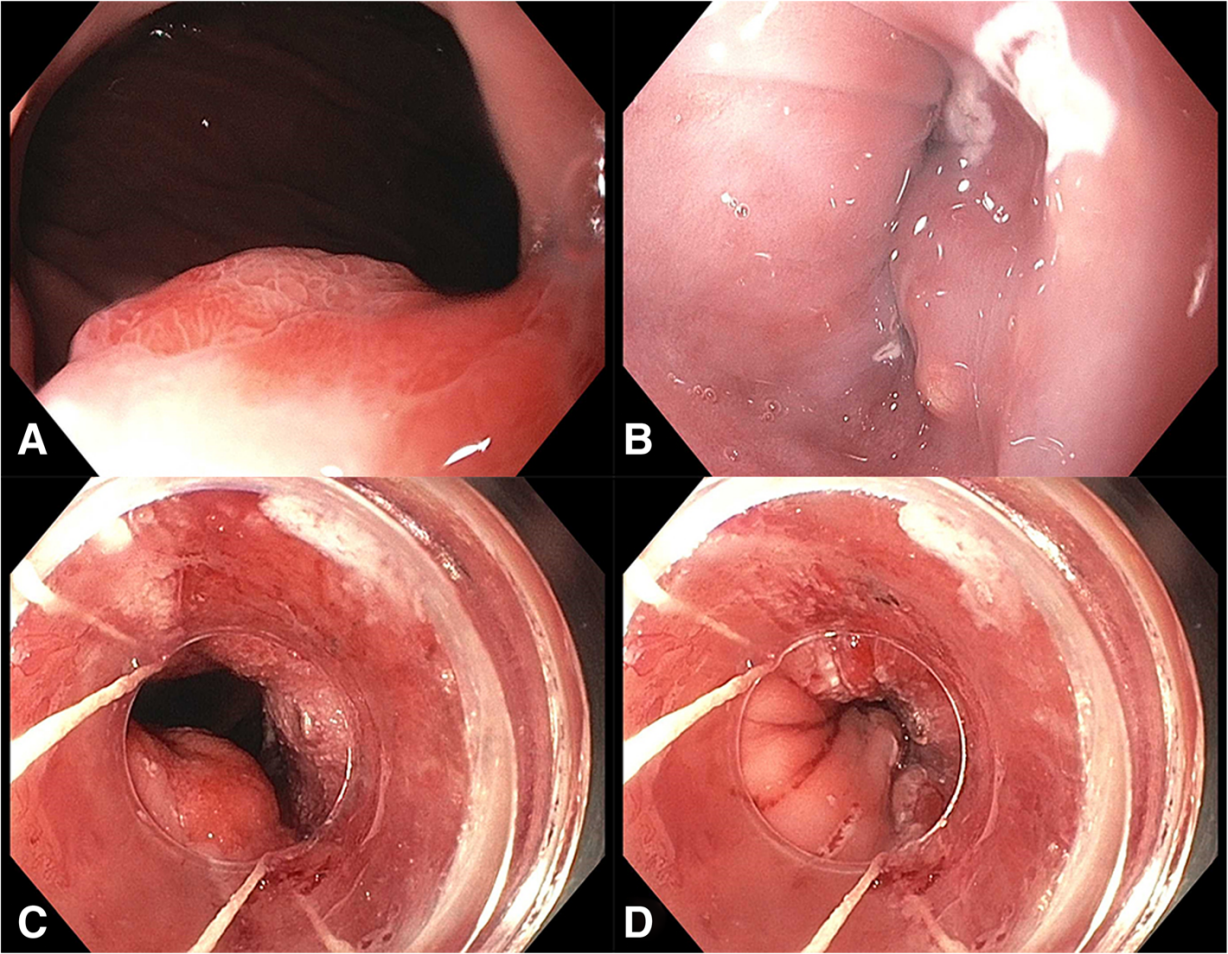
Endoscopy. **a** Mass view from stomach. **b** Mass view from esophagus. **c** Post-resection view when esophagus was dilated. **d** Post-resection view when esophagus was constricted

A 70-year-old Caucasian male with past medical history of hypertension and hyperlipidemia managed with aspirin presented in the emergency room with the complaints of increasing fatigue, shortness of breath and coffee ground stool. An ulcer was found during emergency esophagogastroduodenal endoscopy. He was managed conservatively with Protonix and secession of aspirin. He returned two months later for follow-up esophagogastroduodenal endoscopy. A single 25 mm submucosal nodule was found at the gastroesophageal junction 40 cm from the incisor and appeared to extend 2 cm up into the esophagus (Fig. 1). There was no Barrett esophagus or mucosal disease identified. A biopsy was taken and a diagnosis of adenocarcinoma was rendered. An endoscopic mucosal resection was subsequently conducted. Patient is disease-free for seven months since resection.

### Histopathology

**Fig. 2:**
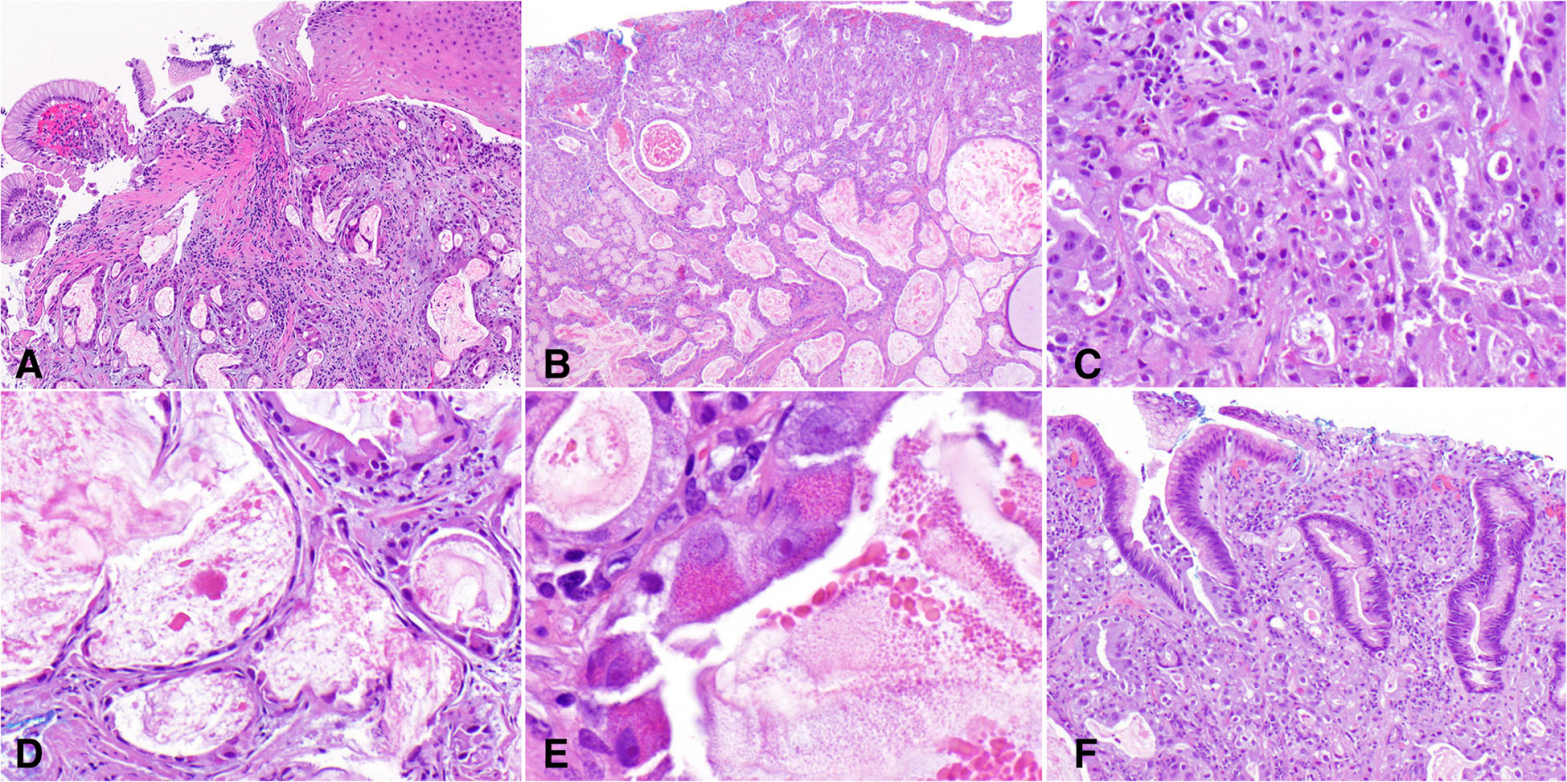
Histopathology. **a** Biopsy (10x). **b**-**f** endoscopic resection: **b** Low power view (4x) showing poorly-differentiated area on the upper half and well-differentiated cystic area on the lower half. **c** Poorly-differentiated area (20x): tumor cells formed small glands and solid growth. **d** Well-differentiated area (20x): bland-appearing cystically dilated glands contained granular eosinophilic luminal secretion. **e** Cytomorphology of the tumor cells (60x): cytoplasm of tumor cells contained eosinophilic coarse granules. **f** Overlying squamous-columnar junction mucosa with reactive changes. No intestinal metaplasia was identified (10x)

The biopsy showed proliferation of haphazard and angulated glands with focal crowding accompanied by desmoplastic stroma underlying squamous-columnar junctional mucosa (Fig. 2a). A diagnosis of adenocarcinoma was made.

The endoscopic mucosal resection specimen demonstrated that the adenocarcinoma invaded into submucosa (Fig. 2b). The tumor showed various levels of differentiation from poorly-differentiated area composed of solid tumor growth and small glands (Fig. 2c) to well-differentiated area composed of cystically dilated glands with attenuated epithelial lining (Fig. 2d). Eosinophilic secretion with focal crystallization was present in the lumina of majority of the glands. The distribution was in such a way that poorly-differentiated area was situated directly below the epithelium and well-differentiated component in the deep portion with differentiation progressing in a gradient fashion.

Despite of various differentiation, tumor cells in different areas showed similar cytomorphology (Fig. 2e). They had abundant cytoplasm (low nuclear: cytoplasm ratio) containing eosinophilic coarse granules and centrally located nuclei, reminiscent of Paneth cells or gastrointestinal neuroendocrine cells. The texture of the cytoplasmic granules was similar to luminal secretion, suggestive of active secretion. The tumor cells in the poorly-differentiated area displayed frankly malignant cytology with pleomorphic nuclei and prominent cherry-red nucleoli. In contrast, the tumor cells in the well-differentiated area had much blander cytology. There was no mitosis or necrosis appreciated.

The mucosa was focally eroded with focal reparative and regenerative changes characterized by pseudostratified columnar cells confined within the crypts (Fig. 2f). The adenocarcinoma focally colonized glandular epithelium. However, no Paneth cell metaplasia was identified. There were no goblet cells as evidence of Barrett esophagus. The adjacent gastric cardiac mucosa showed reactive foveolar hyperplasia and dilated fundic glands with proton pump inhibitor therapy effect.

**Fig. 3:**
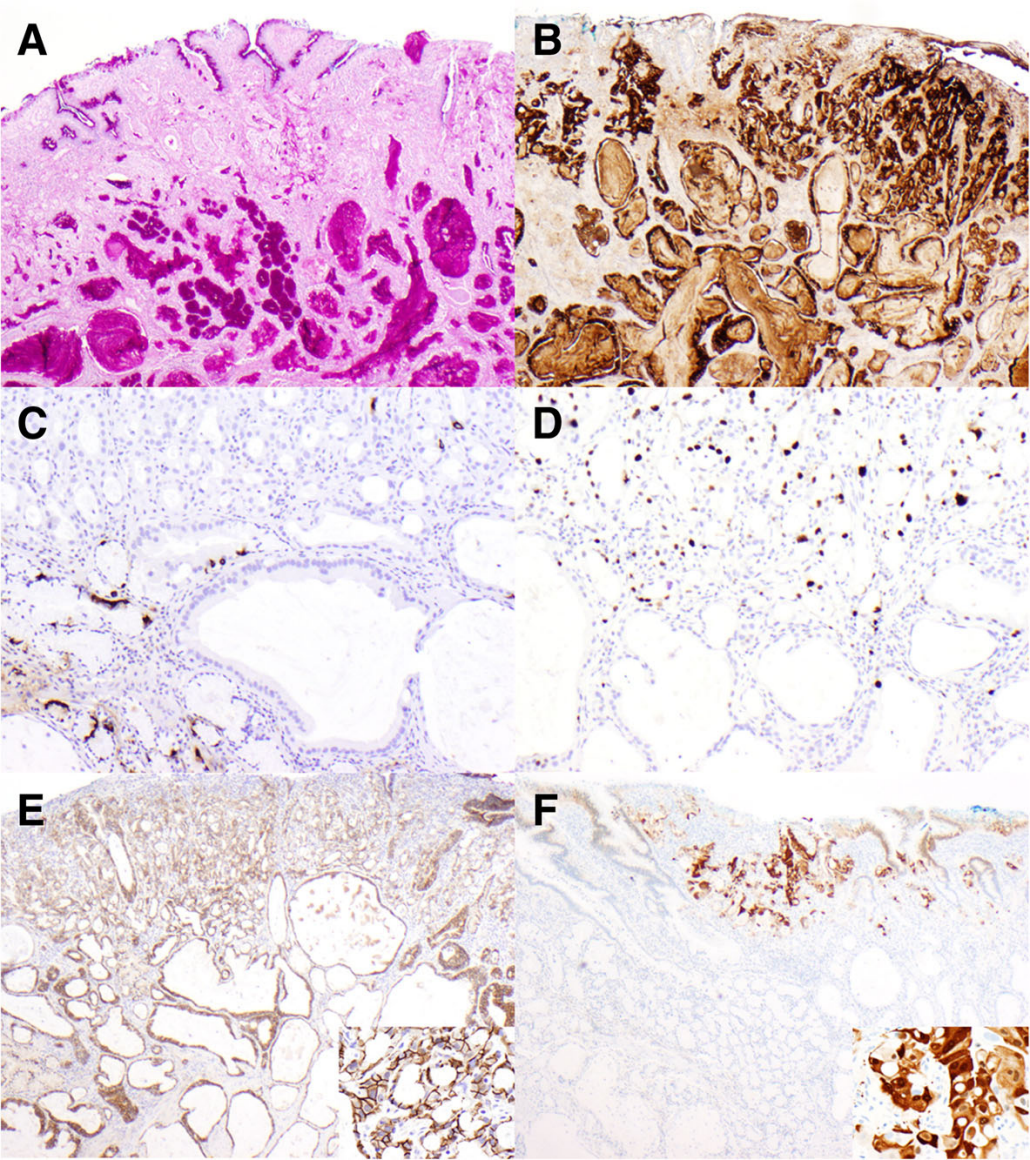
Special and immunohistochemical stains. **a** Periodic acid–Schiff with diastase digestion (4x): diffusely and strongly positive in tumor cells and secretion. **b** Lysozyme (4x): diffusely and strongly positive in tumor cells and secretion. **c** Chromogranin (10x): highlighted residual normal neuroendocrine cells. **d** Ki67 (10x): approximately 40% tumor cells in the poorly-differentiated area (upper half) were positive and only rare positivity was detected in the well-differentiated area (lower half). **e** beta- catenin (4x) (inset: high magnification (40x) of beta-catenin): membranous stain in all the tumor cells regardless of differentiation. **f** Her-2/neu (4x) (inset: high magnification (40x) of Her-2/neu in the poorly-differentiated area): nuclear and cytoplasmic stain in the poorly-differentiated area and absence of stain in the well-differentiated area

To further characterize the cellular origin, special and immunohistochemical stains were performed. Both the cytoplasm of tumor cells and luminal secretion were diffusely and strongly positive for lysozyme immunohistochemical stain (Fig. 3b) and Periodic acid–Schiff with diastase digestion (Fig. 3a). Synaptophysin and chromogranin immunohistochemical stains highlighted scattered entrapped neuroendocrine cells while negative in tumor cells (Fig. 3c). The cytomorphology in combination with strong immunoreactivity with lysozyme antibody and absence of neuroendocrine differentiation support a Paneth cell differentiation. Ki-67 (Fig. 3d) and p53 (data not shown) showed similar staining pattern with positive stain in approximately 40% nuclei of tumor cells in poorly-differentiated area and only rare positivity in well-differentiate area. Mammoglobin was negative in the tumor cells (data not shown). Beta-catenin immunohistochemical stain showed membranous stain in the tumor cells (Fig. 3e). Her-2/neu immunohistochemical stain demonstrated nuclear and cytoplasmic staining in the poorly-differentiated area and was completely negative in the well-differentiated area (Fig. 3f).

## Discussion and conclusion

Paneth cells are present in chronic non-neoplastic conditions such as inflammatory bowel diseases [3] as well as neoplastic conditions such as adenoma or carcinoma [4–6]. Rarely, tumors composed predominantly of neoplastic Paneth cells were reported. Although neoplastic Paneth cells have been described in adenomatous conditions [7–9], the majority of these tumors were adenocarcinomas of gastrointestinal tract. They were mostly reported in stomach [10–16] and colon [6, 8, 17–20] but have also been reported in small intestine [21, 22], duodenal ampulla [23], gallbladder [24] and Meckel’s diverticulum [25]. Exceptions were two reports of extra-gastrointestinal Paneth cell-containing tumors: one was an intestinal type nonurachal adenocarcinoma of urinary bladder [26] and the other were three breast carcinomas associated with microglandular adenosis [27].

Here we described a case of gastroesophageal adenocarcinoma composed entirely of cytologically malignant Paneth cells evidenced by diffuse and strong reactivity to lysozyme antibody in both tumor cells and luminal secretion. The possible neuroendocrine differentiation, which may present with similar morphology, was ruled out by negative synaptophysin and chromogranin immunostains. To our best knowledge, it is the first such case reported in gastroesophageal junction.

There were two unique morphological features in the presented case. One was zonal distribution of areas with various levels of differentiation. The well-differentiated bland-appearing neoplastic glands mimicked dilated fundic glands that may lead to an erroneous diagnosis of fundic gland polyp or proton pump therapy effect if only the well-differentiated component of the lesion is sampled on biopsy. The other feature was the cystically dilated glands with secretion, superficially resembling secretory carcinoma. However, mammoglobin, a protein characteristically positive in secretory carcinoma, was negative.

The etiology of Paneth cell carcinoma is unknown. It is logical to assume it may develop from a pre-neoplastic condition harboring Paneth cells such as intestinal metaplasia of the stomach [28] or Barrett esophagus [29]. The presence of Paneth cell in these conditions is not necessarily antimicrobial. One study demonstrated that *K-ras* mutation and loss of heterozygosity of microsatellite markers were more frequently detected in the colonic mucosa with Paneth cell metaplasia in comparison to that with no Paneth cell metaplasia [30]. Therefore, Paneth cell metaplasia likely represents a pre-neoplastic mucosa at least in colon. However, the lack of Barrett esophagus and Paneth cell metaplasia in our case does not support that Paneth carcinoma develops from Paneth cell metaplasia and or Barrett esophagus. A recent study suggested that the molecular events associated with neoplastic Paneth cells may be different from Paneth cell metaplasia in pre-neoplastic conditions. All neoplastic Paneth cells in their study showed nuclear beta-catenin reactivity, an evidence of activated Apc/beta-catenin/Tcf pathway, while non-neoplastic Paneth cells showed membranous staining pattern [31]. The tumor cells in the presented case did not show any nuclear beta-catenin positivity and therefore may have arisen from a different etiology. The seemingly “maturing” pattern seems to suggest a stem cell origin.

The clinical implication of Paneth cell lesion has not been carefully examined. Paneth cell metaplasia in pre-neoplastic lesions seems to render a favorable prognosis. The presence of Paneth cell metaplasia in Barrett esophagus was associated with less disease regression [29]. Similarly, Paneth cells in distal colorectal adenomas was inversely associated with synchronous advanced adenoma and carcinoma [32]. No long-term follow-up is available for Paneth cell carcinoma in gastrointestinal system although the report of Paneth cell-containing breast carcinomas demonstrated that two of three patients survived for three years with no evidence of disease and one patient died of her disease at 34 months [27]. More studies are needed to demonstrate the prognosis of Paneth cell carcinoma in gastrointestinal system.

There is no specific treatment for Paneth cell carcinoma. As aforementioned, activated Apc/beta-catenin/Tcf pathway was suggested in neoplastic Paneth cells. Human defensin 5, a target of gene of Apc/beta-catenin/Tcf pathway, was newly discovered to be a specific marker of Paneth cells [31] and therefore a potentially drugable target. Interestingly, human defensin 5 expression was also present in a subset of colonic epithelial neoplasms without morphologic evidence of Paneth cell differentiation [31]. Therefore, a therapy targeting at human defensin 5 may have wider applications in addition to treating Paneth cell carcinoma. A peculiar Her-2/neu staining pattern was observed in the presented tumor: the poorly-differentiated area showed strong nuclear and cytoplasmic reactivity and the well-differentiated area had no stain at all. Although considered as negative by current standard [33], this staining pattern may have clinical implications. For example, a significant proportion (30%) of colorectal cancer was found to have cytoplasmic Her-2/neu expression which has been associated with a poor prognosis [34].

In conclusion, we presented the first case of Paneth cell carcinoma at gastroesophageal junction with a potentially misleading morphology. Larger studies are needed to illustrate the etiology, pathogenesis, clinical course, prognosis and treatment.
